# ﻿Identification of *Sindiplozoon coreius* (Monogenea, Diplozoidae) and morphological characteristics of the various developmental stages

**DOI:** 10.3897/zookeys.1258.162589

**Published:** 2025-11-03

**Authors:** Lu Shen, Zhuo-Yu Zhao, Ting Jiang, Jun-Dong Xu, Han-Ji Tian, Yao-Yue He, Ting Jia, Wei-Jiang Xu, Fei-Yan Meng, Li-Xian Fan

**Affiliations:** 1 School of Life Science, Yunnan Normal University, Kunming 650500, China Yunnan Normal University Kunming China; 2 Engineering Research Center of Sustainable Development and Utilization of Biomass Energy, Ministry of Education, Yunnan Normal University, Kunming 650500, China Yunnan Normal University Kunming China; 3 Yunnan Key Laboratory of Biomass Energy and Environmental Biotechnology, Yunnan Normal University, Kunming 650500, China Yunnan Normal University Kunming China

**Keywords:** Different developmental period, diplozoids, ectoparasitic, ITS2, life cycle, ontogenetic development

## Abstract

Diplozoids are ectoparasites that mainly infect the gills of freshwater fish. While the life cycles of *Eudiplozoon nipponicum* (Goto, 1891) Khotenovsky, 1985 and some *Paradiplozoon* Achmerov, 1974 species are documented, *Sindiplozoon* Khotenovsky, 1981, development remains unclear. During a survey of fish parasites, diplozoids were collected from the predatory carp, *Chanodichthys erythropterus* Basilewsky, 1855, in the Lancang River and cultured Kanglang fish, *Anabarilius graham* Regan, 1908, in Kunming. Morphological and molecular methods confirmed all specimens as *Sindiplozoon coreius* Cao, 2022, and five developmental stages with their typical features were observed through morphological observations: oval egg with filament; ciliated oncomiracidium with hooks and one pair of clamps; diporpa with additional clamps; X-shaped juvenile with developing clamps; and adult with complete clamps and mature reproductive system. Morphometric analysis showed the central hook grew significantly during the transition from oncomiracidium to diporpa (*p* < 0.001). The buccal sucker, pharynx, and body length increased notably from juvenile to adult (*p* < 0.001). Clamps developed steadily throughout the life cycle, reaching maximum maturity at the adult stage. This is the first detailed description of *S. coreius* development, confirming species identity and expanding its known host range and distribution.

﻿**Graphical Abstract**

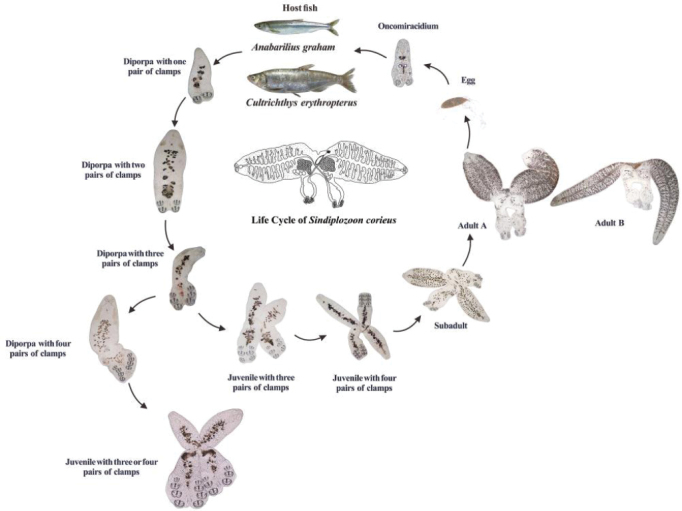

## ﻿Introduction

Species in Diplozoidae Palombi, 1949 (Monogenoidea: Mazocraeidea) are typically found on gills of cypriniform fishes (Přikrylová et al. 2018; Wu et al. 2000; Benovics et al. 2021). Diplozoids are known to cause hyperaemia, haemorrhage, and atrophy in fish gills, which, in turn, adversely affects the respiratory function of the host fish (Lu et al. 2008; İnnal et al. 2020; Cao et al. 2024). Adult diplozoids exhibit a distinctive morphology, forming a permanent X-shaped structure resulting from the fusion of two individual worms. Numerous studies have focused on the morphology (Heckmann et al. 2012; Civáňová et al. 2013; Konstanzová et al. 2017; Fan et al. 2018), and phylogeny (Gao et al. 2006; Zhang et al. 2018; Dos Santos and Avenant-Oldewage 2020; Hao et al. 2022; Nejat et al. 2023; Vorel et al. 2023; Dos Santos and Avenant-Oldewage 2024) of diplozoids.

Diplozoidae comprises seven accepted genera with species assigned to five of them occurring in China (Jiang et al. 1989; Wu et al. 2000; Gao et al. 2006; Wang et al. 2015; Fan et al. 2018; Huang et al. 2023): *Diplozoon* Nordmann, 1832; *Paradiplozoon* Achmerov, 1974; *Inustiatus* Khotenovsky, 1978; *Sindiplozoon* Khotenovsky, 1981 and *Eudiplozoon* Khotenovsky, 1985. Among them, seven species of *Sindiplozoon* have been reported in China: *S. diplodiscus* Khotenovsky, 1978, *S. fujianensis* Jiang, 1989, *S. hunanensis* Yao, 1997, *S. xenocypris* Jiang & Wu, 1983, *S. ctenopharyngodoni* Ling, 1973 (Chen and Li 1973), *S. strelkowi* Khotenovsky, 1978, and *S. coreius* Cao, 2022. Notably, *S. coreius* was firstly described on the gills of *Coreius guichenoti* Sauvage & Dabry, 1874 from the Yangtze River in 2020 (Cao et al. 2022) and was subsequently reported from cultured *Percocypris pingi* Tchang, 1930, *Schizothorax prenanti* Tchang, 1930, and *Procypris merus* Lin, 1933 in the Xijiang River system (Cao et al. 2024). In previous studies, the life cycle of *E. nipponicum* (Hodová and Sonnek 2009; Hodová et al. 2010; Valigurová et al. 2011) and *Paradiplozoon* spp. (Pečínková et al. 2007; Avenant-Oldewage and Milne 2014; Zhao 2020) have been described in detail, but the developmental stages of *Sindiplozoon* remain unknown. Therefore, documenting its developmental stages is essential for clarifying host specificity and parasitic strategy, and also provides a basis for comparative studies with other diplozoids to better understand their evolutionary and ecological adaptations.

During the survey of fish parasites, a species of diplozoid, identified as *Sindiplozoon coreius*, was detected in the predatory carp, *Chanodichthys erythropterus* Basilewsky, 1855 and the Kanglang fish, *Anabarilius graham* Regan, 1908, both belonging to Xenocyprididae (Cypriniformes). Previous studies have reported parasites in predatory carp (Nie et al. 2000; Yao 2001; Weng et al. 2023), but no diplozoids have been recorded from this host. Adult specimens from predatory carp represent a new locality record for *S. coreius*, and diplozoids at various developmental stages were observed in Kanglang fish. This study also documents the ontogenetic development of *S. coreius* for the first time, contributing to a better understanding of its host specificity and life history.

## ﻿Material and methods

### ﻿Sample collection

**Figure 1.**
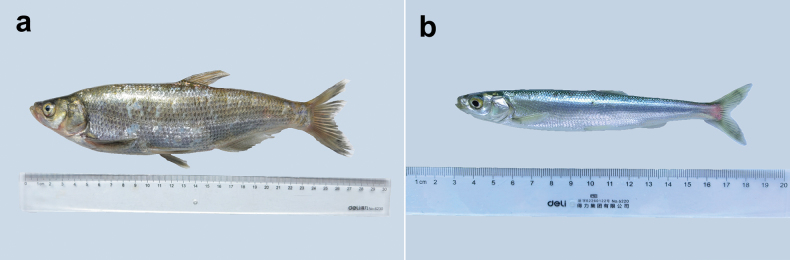
Host fish. a. *Chanodichthys erythropterus* Basilewsky, 1855; b. *Anabarilius graham* Regan, 1908.

Predatory carp (Fig. 1A) were collected from the Lancang River (Nanjian River basin) in July 2024, while Kanglang fish (Fig. 1B) were collected from a fish farm in Kunming in November 2024. Hosts were first anesthetized and euthanized, after which the gill was excised and examined under a dissecting microscope. Individual diplozoids were removed by using a fine brush and an anatomical needle. Diplozoids were water-mounted, photographed under a compound microscope (Olympus CX-41), and their whole body, oral sucker, pharynx, central hooks and clamps were subsequently observed and measured as straight-line distances between the extreme ends using Capture 3.0 (Tucsen Photonics Co., Ltd). The illustrations are taken with a light microscope and with the aid of a drawing digital board (Wacom Intous Pro), then processed on a computer using Photoshop CS4.0 (Adobe, San Jose, CA, USA).

### ﻿Preparation of stained specimens

Diplozoid specimens preserved in ethanol were retrieved using a fine brush and gradually rehydrated through an ethanol gradient. The specimens were sequentially transferred into Petri dishes containing 90%, 80%, 70%, and 50% ethanol, each for 3 h. Following this, the specimens were transferred to Petri dishes containing distilled water and left overnight. Once the diplozoids had regained sufficient flexibility, they were placed on water-filled glass slides. Coverslips were gently applied using forceps and fixed at the corners with nail polish. The slides were then placed in Petri dishes containing Bouin’s solution for fixation for 5–7 h. After fixation, the nail polish was carefully removed with a dissecting needle, and the coverslips were gently lifted to avoid tearing the specimens. The parasites were then transferred to clean water and washed for 1 h, with the water replaced frequently during the process.

The washed specimens were transferred into Petri dishes containing alum carmine staining solution and stained for 12 h. During the staining process, specimens were briefly removed using a fine brush, rinsed with water, and examined under a microscope to monitor staining progress. After staining, the specimens were rinsed several times with distilled water. Differentiation was carried out under a microscope using acid alcohol for 20–30 s until internal structures became clearly visible. If the specimens appeared under-stained after differentiation, restaining with alum carmine solution was performed. Following differentiation, the specimens were dehydrated through a graded ethanol series of 70%, 80%, 90%, 100% I, and 100% II, each for 2 h. They were then treated with a xylene: ethanol mixture (1:1, v/v) for 1 h and cleared in cedarwood oil for 12 h. The cedarwood oil was subsequently removed with xylene. Finally, the specimens were mounted on clean glass slides using neutral balsam and labeled accordingly (Bai et al. 2014; Bai 2015; Meng 2017).

### ﻿Molecular analysis of diplozoids

Genomic DNA was extracted from the parasites using the TIANamp Micro DNA Kit (Beijing, China), following the manufacturer’s protocol. The ITS2 region of the genomic DNA was amplified using universal primers (Matejusová et al. 2001):

D (5′–GGCTYRYGGNGTCGATGAAGAACGCAG–3′)

B1 (5′–GCCGGATCCGAATCCTGGTTAGTTTCTTTTCC–3′)

Polymerase Chain Reaction (PCR) amplification was performed in a 50 μL reaction mixture containing 2 μL DNA template, 19 μL reaction buffer (including dNTPs, 10 × buffer, and Taq polymerase), 2 μL of each primer, and 25 μL of double-distilled water. The thermal cycling conditions were as follows: an initial denaturation step at 90 °C for 10 min, followed by 30 cycles of denaturation at 95 °C for 30 s, annealing at 55 °C for 30 s, and extension at 72 °C for 75 s, with a final extension step at 72 °C for 10 min.

PCR products were visualized on 1% agarose gels stained with GoodView (Tanon). DNA fragments were sequenced, and the resulting sequences were submitted to the National Center for Biotechnology Information (NCBI) database for BLAST searches. Eighteen diplozoid sequences were selected from NCBI, and a phylogenetic analysis was conducted incorporating these species along with those collected in the present study (Suppl. material 1: table S1). Base composition data, parsimony and nucleotide substitutions between pairwise distances (Kimura 2-parameter) were estimated using MEGA 6.0 and BLAST from NCBI. The robustness of topologies was assessed by 1000 bootstrap replicates. *Neoheterobothrium hirame* Ogawa, 1999 (Monogenoidea, Diclidophoridae) was used as the outgroup.

### ﻿Statistical analysis

The statistical analysis of experimental data was conducted using the SPSS 21.0 software package. To examine the relationship between structural size and different life cycle stages, a one-way repeated measures ANOVA (Analysis of variance) was employed. The LSD (Least Significant Difference) multiple comparison was performed to further investigate which groups exhibit significant differences. Results are presented as mean ± SE (standard error of the mean), with *p* < 0.05 (Probability value) indicating a significant difference and *p* < 0.01 indicating a highly significant difference. In the statistical analysis, stage 1 through 7 represent the following developmental stages: Oncomiracidium (Stage 1), diporpa with one pair of clamps (Stage 2), diporpa with two pairs of clamps (Stage 3), diporpa with three pairs of clamps (Stage 4), juvenile with three pairs of clamps (Stage 5), juvenile with four pairs of clamps (Stage 6), and adult (Stage 7).

### ﻿Abbreviations

**LSD**: Least significant difference; **ANOVA**: Analysis of variance; **SE**: Standard error of the mean; **ME**: Minimum evolution; **F**: F-ratio (F statistic); **p**: P-value (Probability value); **μm**: micrometer.

## ﻿Results

### ﻿Collection results

In this study, 8 adult diplozoid specimens were collected from a single host fish of the predatory carp from the Nanjian River basin of the Lancang River. Additionally, 91 specimens representing various developmental stages, including two eggs, were collected from 6 Kanglang fish from a fish farm in Kunming (Suppl. material 1: table S2).

### ﻿Morphological identification

The diplozoid specimens collected from the predatory carp and Kanglang fish exhibited highly similar morphological characteristics. The body surfaces are smooth, lacking folds; the testes are composed of five lobules; the eggs are oval in shape and equipped with polar filaments. Additionally, a disc-shaped muscular thickening is present posterior to the copulatory union region.

**Figure 2.**
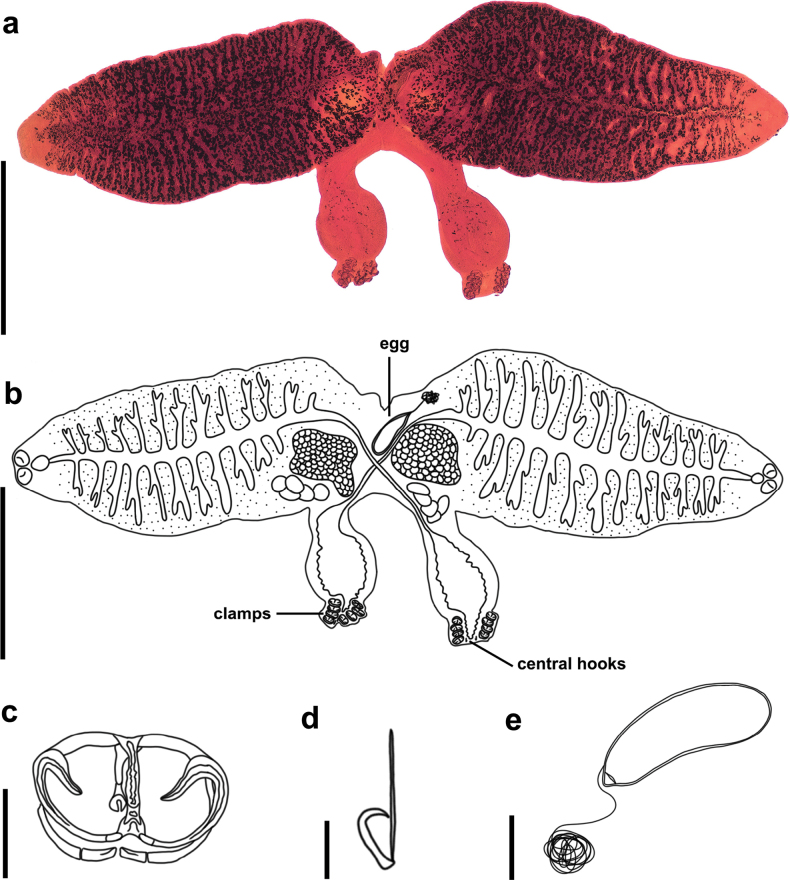
Stained specimen and hand-drawn ink drawing of *Sindiplozoon coreius* Cao, 2022. a. Stained specimen; b. Adult; c. Clamp; d. Central hooks; e. Egg. Scale bars: 1 mm (a, b), 50 μm (c), 20 μm (d), 100 μm (e).

Both of them lack round glands in the anterior region of the buccal suckers, display a widened area with a disciform structure between the posterior and reproductive fusion areas, and have no special lobed enlargement as observed in *Eudiplozoon nipponicum* (Goto, 1891) Khotenovsky, 1985 (Fig. 2A). Owing to our specimens having these unique morphological features, they were identified as a species of *Sindiplozoon* (see Wu et al. 2000).

The adult specimens measured an average length of 5226 ± 304 μm (*N* = 7) (Suppl. material 1: table S3). The intestine was centrally located, extending to the body fusion area (Fig. 2A). The intestine lacks branches in the body fusion area, and long branches extend from the widened area to the first clamp. The vitelline follicles of specimens are numerous and located at the anterior part of the body. Ovary single, ovoid in outline, located in the anterior part of the fusion area. Testis single, composed of 5 ovoid subcomponents, posterior to the ovary. The gonads are distributed in the anterior part of the body fusion area and around the genital organs. Eggs elliptical with long, curly filament attached to operculum, 333 ± 35 μm (*N* = 2) ×108 ± 8 μm (*N* = 2) (Fig. 2D).

Four pairs of clamps and one pair of central hooks are present on the haptors. Clamp I, smallest, 67 ± 1 μm (*N* = 3) × 94 ± 3 μm (*N* = 3). Clamp II 64 ± 1 μm (*N* = 3) × 105 ± 2 μm (*N* = 3). Clamp III, largest, 64 ± 1 μm (*N* = 3) × 108 ± 2 μm (*N* = 3). Clamp IV 61 ± 1 μm (*N* = 3) × 94 ± 3 μm (*N* = 3) (Suppl. material 1: table S3). The clamps were composed of sclerotized structures. The median sclerite was U-shaped, with a thickened anterior end forming a trapezoid outgrowth (Fig. 2B). The anterior clamp jaw consists of two curved sclerites, while the posterior clamp jaw comprised medial and lateral parts. Both the anterior arch of the anterior clamp jaw and the medial part of the posterior jaw lacked cross-striation. The central hooks, located between the terminal protrusion of the haptor and the first pair of clamps are formed by a handle and a sickle through a connection. The sickle was curved toward the handle and had a winged end that bent toward the connection. Central hook crochet en fléau 24 ± 1 μm (*N* = 3), handle 50 ± 1 μm (*N* = 3) (Suppl. material 1: table S3) (Fig. 2C).

### ﻿Molecular analysis

BLASTN analysis of the ITS2 sequences amplified from the diplozoid specimens collected from predatory carp (829 bp) and Kanglang fish (811 bp) both revealed 99.58% similarity to a sequence ascribed to *Sindiplozoon coreius* (GenBank No. MW992745, 721bp). The ITS2 sequences were deposited in GenBank under the following accession numbers: PQ684283 and PQ684284.

**Figure 3.**
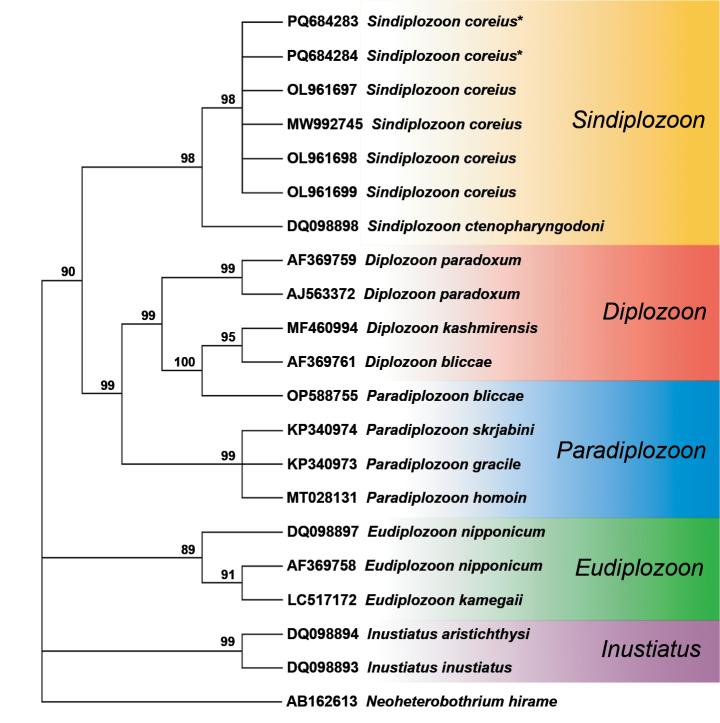
The rooted condensed tree (with 72% cut-off value) based on the ME analysis method. The monogenean *Neoheterobothrium hirame* Ogawa, 1999 was used as the outgroup. *New sequence obtained in the present study.

According to the rooted condensed tree (with 72% cut-off value) based on the ME (Minimum Evolution) analysis method, our sequences were positioned in a clade comprising other *Sindiplozoon* spp. (Fig. 3). All *S. coreius* sequences reovered in a clade sister to *S. ctenopharyngodoni* Ling, 1973 (GenBank No. DQ098898) from this study clustered in a single branch representing *S. coreius*, forming a sister group with *Paradiplozoon* and *Diplozoon* species (Fig. 3). Comparison with 20 previously submitted diplozoid sequences (Suppl. material 1: table S4) further validated the homogeneity and genetic similarity of the ITS2 sequences obtained in this study. The closest related sequences were those of *S. coreius* reported from *Coreius guichenoti* in China.

The identification of the parasites from the two host species as *S. coreius* was supported by both morphological characteristics and molecular phylogenetic analyses based on nucleotide sequences.

## ﻿Different developmental stages of *Sindiplozoon coreius*

### ﻿The egg stage of *Sindiplozoon coreius*

**Figure 4.**
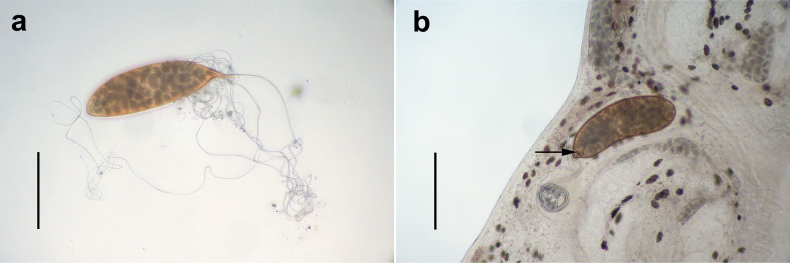
The egg of *Sindiplozoon coreius* Cao, 2022. a. Egg; b. Egg in utero; operculum (arrow). Scale bars: 200 μm (a, b).

The egg is located in the anterior part of the reproductive fusion area of the adult *S. coreius*. It is ovoid in shape, smooth, without any protruding surface. A long, coiled filament is attached to the operculum located at one pole of the egg. In this study, two eggs were observed. One egg was located in utero (Fig. 4B), with its filaments neatly coiled. While the other had already been deposited and its filaments are disordered (Fig. 4A).

### ﻿The oncomiracidium stage of *Sindiplozoon coreius*

**Figure 5.**
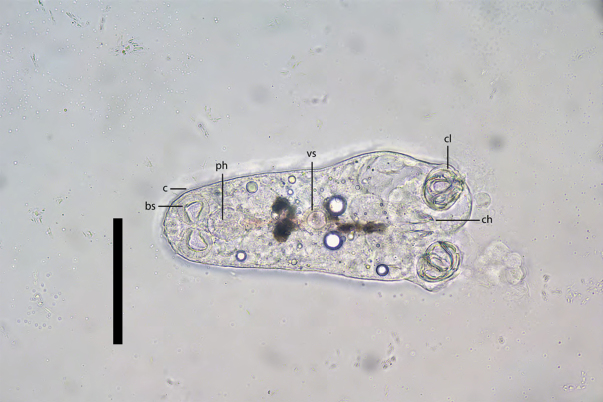
The oncomiracidium of *Sindiplozoon coreius* Cao, 2022. bs: buccal suckers; c: cilia; ph: pharynx; vs: ventral sucker; cl: clamps; ch: central hooks. Scale bar: 100 μm.

The oncomiracidium hatches from the egg and has cilia on the tegument. The anterior part of the oncomiracidium contains an open mouth, paired buccal suckers, and a pharynx situated posterior to the buccal suckers. A circular sucker is positioned centrally on the ventral side of the body. The posterior portion of the worm is equipped with a pair of small central hooks and a pair of bilateral clamps (the first clamp) (Fig. 5).

### ﻿The diporpa stage of *Sindiplozoon coreius*

**Figure 6.**
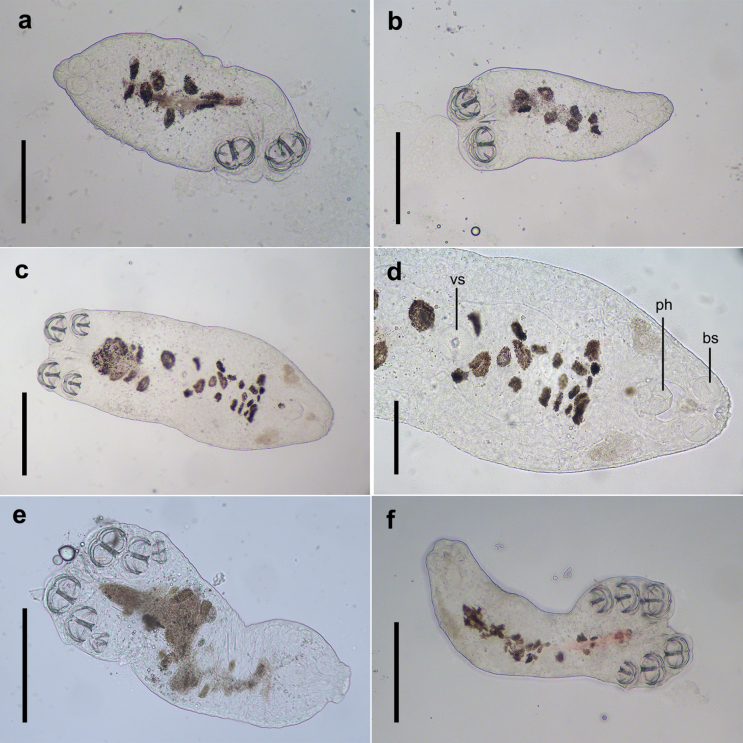
The diporpa of *Sindiplozoon coreius* Cao, 2022 with 1–3 pairs of clamps. a, b. Diporpa of one pair of clamps; c. Diporpa of two pairs of clamps; d. Ventral sucker, pharynx and buccal suckers; e, f. Diporpa of three pairs of clamps. bs: buccal suckers; ph: pharynx; vs: ventral sucker. Scale bars: 200 μm (a–c, e, f); 100 μm (d).

**Figure 7.**
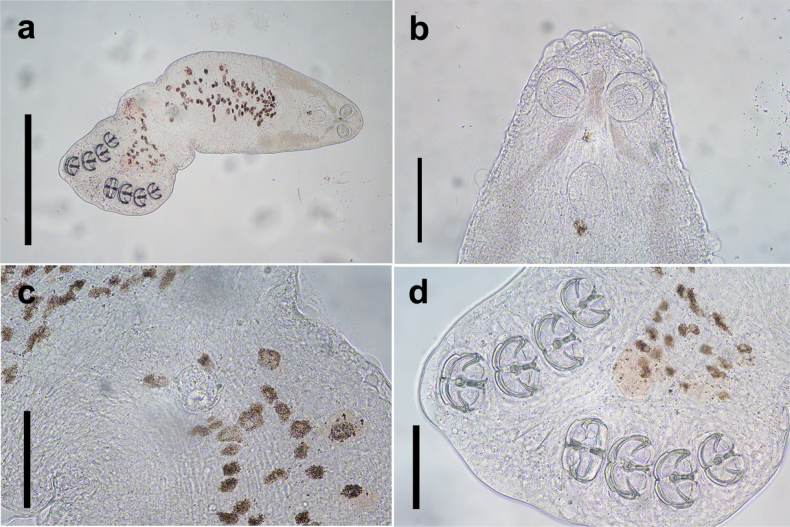
The diporpa of *Sindiplozoon coreius* Cao, 2022 with 4 pairs of clamps. a. Whole body; b. Buccal suckers and pharynx; c. Ventral sucker; d. Haptor. Scale bars: 500 μm (a); 100 μm (b–d).

Once the oncomiracidium attaches to the gill of the host, the cilia are shed, the central hooks stop growing (Fig. 14), and additional clamps begin to develop (Fig. 6). The second and third clamp pairs form alongside the ventral sucker in the haptor, and a protuberance forms on the dorsal surface. Worms that do not pair with another individual at this stage can still develop a fourth clamp pair (Fig. 7A).

### ﻿The juvenile stage of *Sindiplozoon coreius*

**Figure 8.**
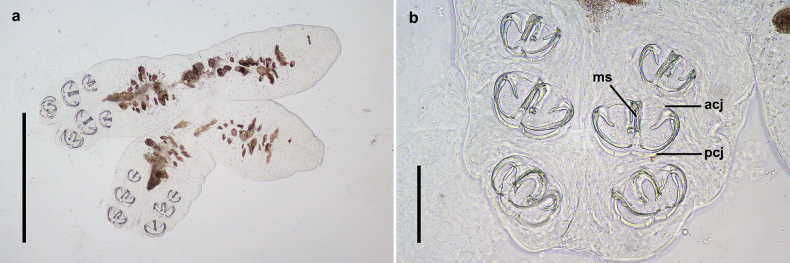
The juvenile of *Sindiplozoon coreius* Cao, 2022 with 3 pairs of clamps. a. Whole body; b. Haptor. ms: median scleritc; acj: anterior clamp jaw; pcj: posterior clamp jaw. Scale bars: 500 μm (a); 100 μm (b).

**Figure 9.**
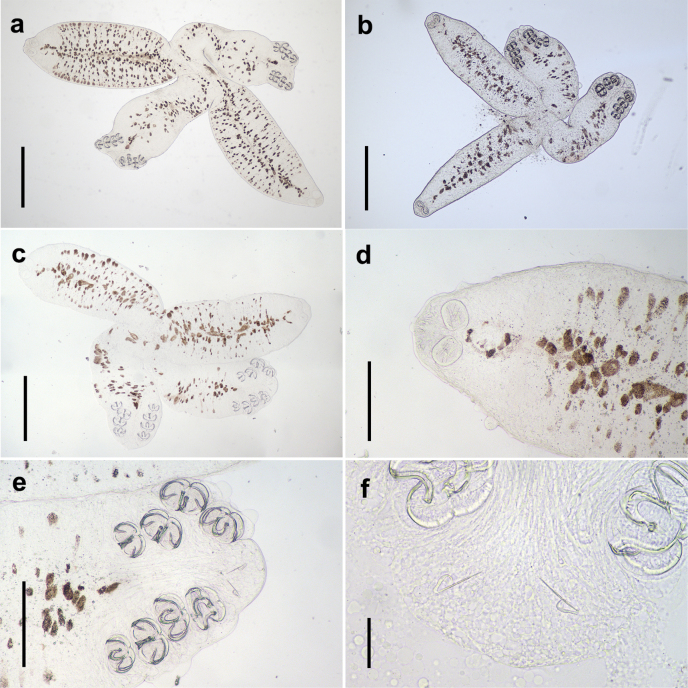
The juvenile of *Sindiplozoon coreius* Cao, 2022 with 4 pairs of clamps. a–c. Whole body of *S. coreius* with 4 pairs of clamps; d. Pharynx and buccal suckers; e. Posterior; f. Central hooks. Scale bars: 500 μm (a–c); 200 μm (d, e); 50 μm (f).

**Figure 10.**
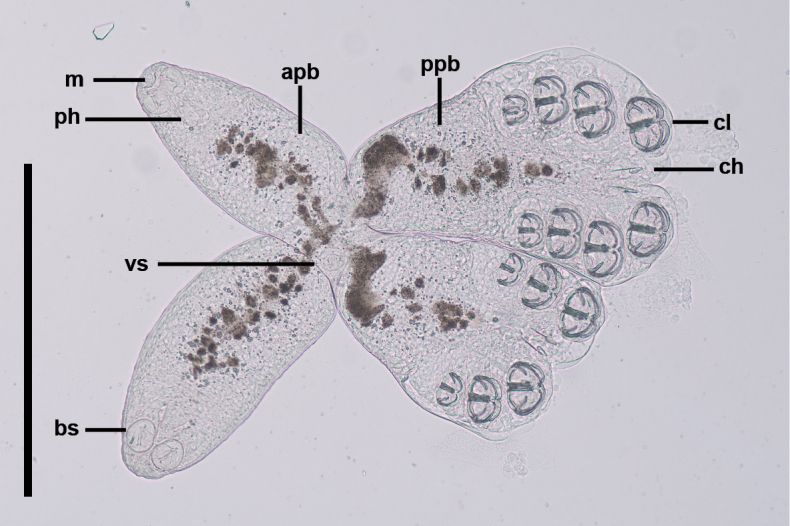
The juvenile of *Sindiplozoon coreius* Cao, 2022. bs: buccal suckers; m: mouth; ph: pharynx; vs: ventral sucker; cl: clamps; ch: central hooks; apb: anterior part of body; ppb: posterior part of body. Scale bar: 500 μm.

**Figure 11.**
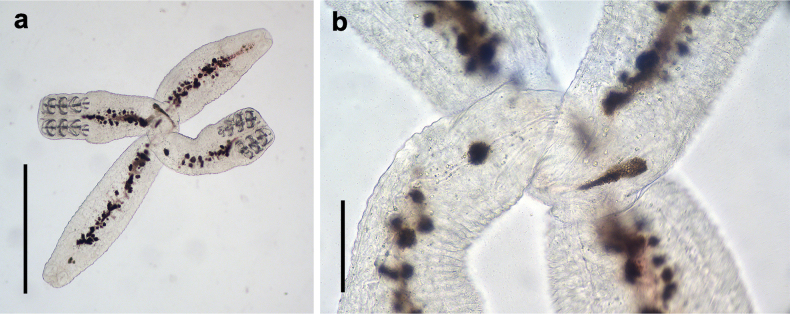
The juvenile of *Sindiplozoon coreius* Cao, 2022. a. Whole body; b. Fusion area. Scale bars: 500 μm (a); 200 μm (b).

During the juvenile stage, pairing is usually initiated after the third clamp pair develops. Two individuals join at the dorsomedian protuberance formed in the diporpa stage and suck onto one another with their ventral suckers (Fig. 8A), with both individuals forming a permanent X-shaped structure (Fig. 9A, B). At this stage, the first and second pairs of clamps are fully developed, while the third pair of clamps remains incomplete (Fig. 9), and the fourth pair begins forming. In this early period, it is common for three pairs of clamps to form an X-shaped permanent pair; however, three pairs of clamps with four pairs of clamps have also been observed (Fig. 10). The juvenile worm is relatively small, with immature internal reproductive genitalia. The fusion area is not yet fully developed, and the ventral sucker remains visible. Our observations show that the fusion areas of two paired worms twist together, but the edges are distinct, indicating incomplete fusion (Fig. 11).

Unpaired worms observed with four clamps suggest that delayed pairing allows for continued clamp development.

### ﻿The adult stage of *Sindiplozoon coreius*

**Figure 12.**
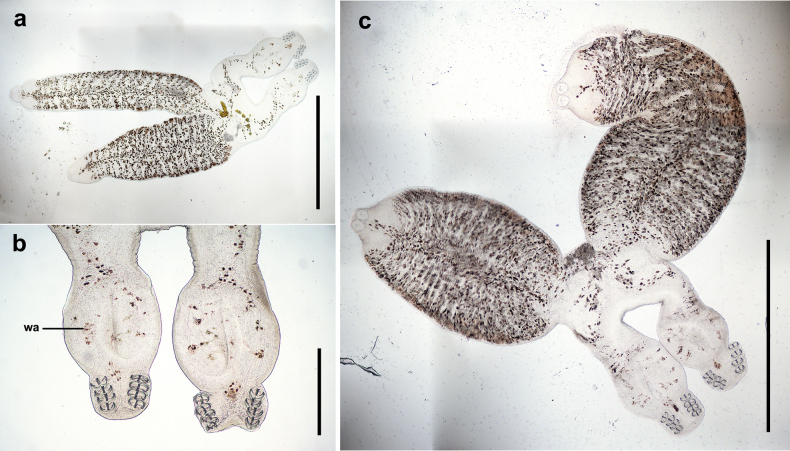
The adult of water-sealed microslides of *Sindiplozoon coreius* Cao, 2022. a. Extended state of the whole body; b. Hindbody of adult; c. Contraction state of the whole body. wa: widened area. Scale bars: 2 mm (a, c); 500 μm (b).

The adult stage of *S. coreius* is characterized by the permanent fusion of two individuals into a distinct X-shape (Fig. 12). The mouth is located on the anterior ventral side, accompanied by a pair of round buccal suckers with horizontal striations on the dorsal side. The pharynx lies posterior to the buccal suckers, and the intestine extends into the fusion area. Between the fusion area and the posterior end, a muscular, nearly circular “Cup-like” (Cao et al. 2022) widened area is present on both the dorsal and ventral surfaces (Fig. 12B).

**Figure 13.**
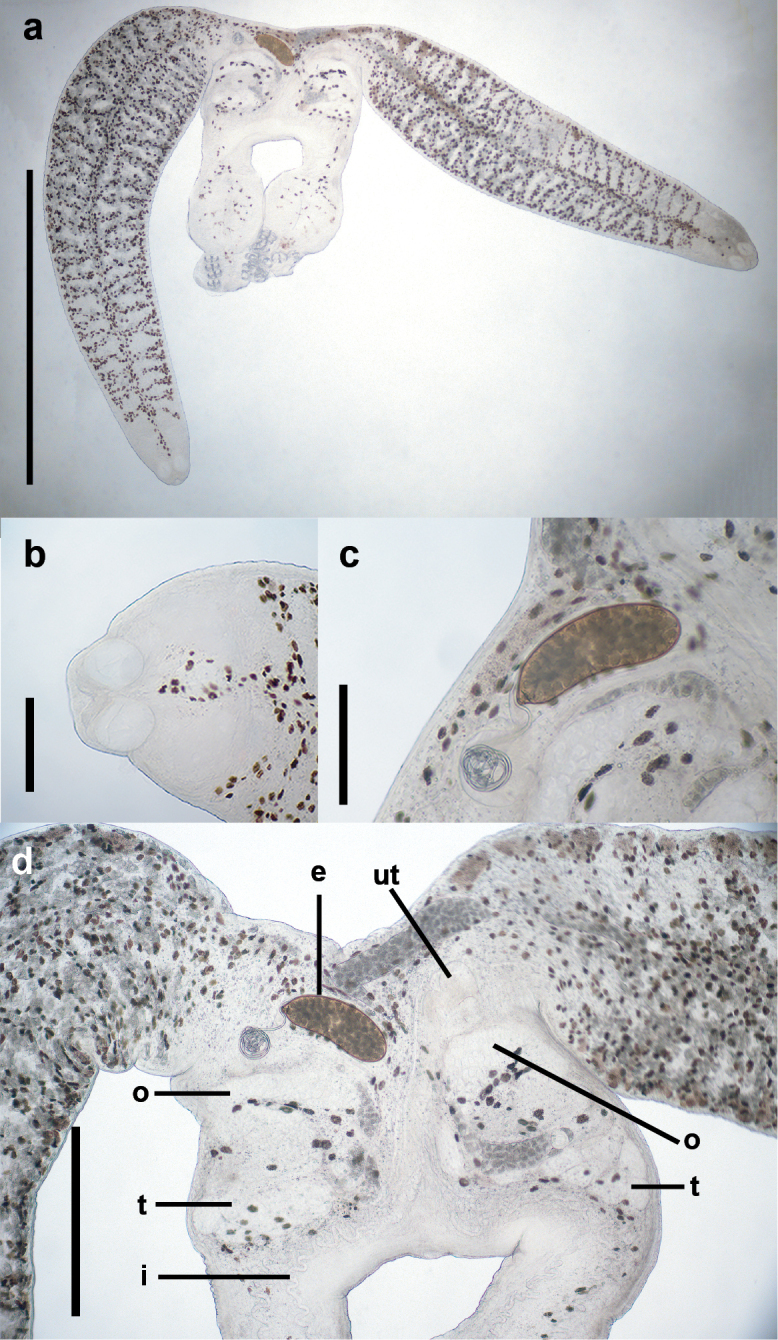
The adult of *Sindiplozoon coreius* Cao, 2022 with egg. a. Whole body; b. Buccal suckers and pharynx; c. Egg; d. Fusion area; e. Egg; ut: uterus; o: ovary; t: testis; i: intestine. Scale bars: 2 mm (a); 200 μm (b); 200 μm (c); 500 μm (d).

The posterior end of the worm features four pairs of clamps and a single pair of central hooks (Fig. 12B). An oval ovary is located in the fusion area anteriorly, and a single testis comprising five subcomponents that are ovoid in outline and located posterior to the ovary (Fig. 13D). The gonads are distributed in the anterior part of the fusion area. The egg, found in the uterus, is oval with a thin, curly filament attached to the operculum. A suture between the operculum and the egg was clearly observed. (Fig. 13B).

**Figure 14.**
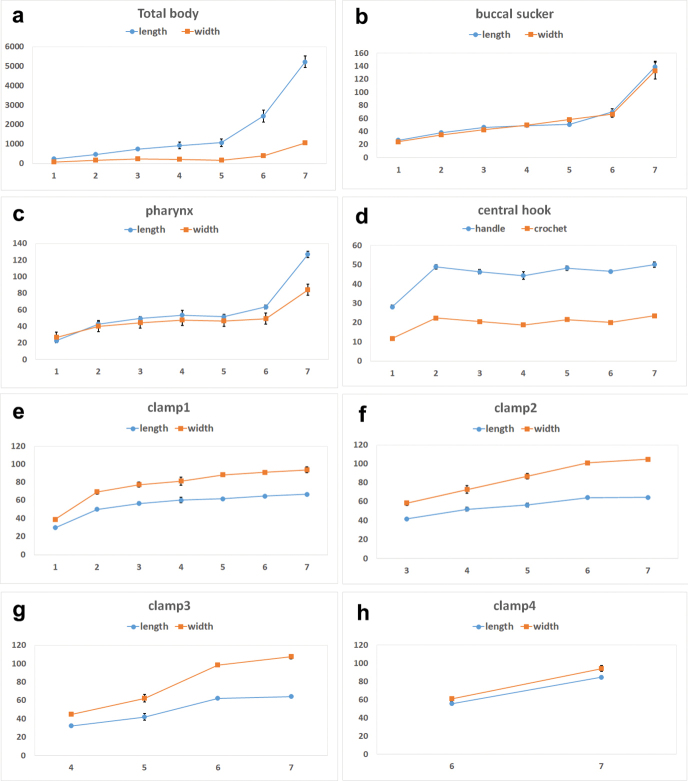
Growth of different structures at different stages life cycle of *Sindiplozoon coreius* Cao, 2022. a. Growth of total body at different stages of the life cycle; b. Growth of buccal sucker at different stages of the life cycle; c. Growth of pharynx at different stages of the life cycle; d. Growth of central hook at different stages of the life cycle; e. Growth of clamp I at different stages of the life cycle; f. Growth of clamp II at different stages of the life cycle; g. Growth of clamp III at different stages of the life cycle; h. Growth of clamp IV at different stages of the life cycle (data of diporpa, which have 4 pairs of clamps, were not used in the figure; buccal suckers and clamps are plotted by means of the average value of the left and right structures. 1. Oncomiracidium, 2. Diporpa with 1 pair of clamps, 3. Diporpa with 2 pairs of clamps, 4. Diporpa with 3 pairs of clamps, 5. Juvenile with 3 pairs of clamps, 6. Juvenile with 4 pairs of clamps, 7. adult).

### ﻿Description of different developmental stages of *Sindiplozoon coreius*

The life cycle of *S. coreius* involves several distinct stages:

- Egg:

- The egg is oval, approximately 333 μm in length and 108 μm in width, with an operculum and a long filament attached to it.

- Oncomiracidium:

- After hatching, the oncomiracidium is ciliated, allowing it to swim freely and locate a host. It has a pair of small central hooks, a single pair of clamps, buccal suckers, a pharynx, and a ventral sucker.

- Diporpa:

- Upon attaching to the gill of the host, cilia are shed, central hooks and ventral sucker develop fully. Additional pairs of clamps (second and third) form sequentially. Unpaired worms can develop a fourth pair of clamps independently.

- Juvenile:

- Worms pair permanently, forming an X-shaped structure. The first and second pairs of clamps are fully developed, while the third pair is incomplete, and the fourth pair is beginning to form. The ventral sucker remains visible, and internal organs are still underdeveloped.

- Adult:

- The fusion of two worms is complete, resulting in a mature X-shaped structure. The fourth pair of clamps is fully developed, and the reproductive system is mature. The adult body length is approximately 21 times that of the oncomiracidium stage.

### ﻿Relationship between different structures and the life cycle of *Sindiplozoon coreius*

In the LSD multiple comparison test, significant increases in both body length and width were observed over time. The ANOVA results show that *F* (F statistic) = 37.959 (*p* = 0.000) for body length and *F* = 23.722 (*p* = 0.000) for body width, indicating significant differences among the groups for both body length and body width. Furthermore, the results of the LSD test provide additional support for this conclusion. The body length difference between stages 6 and 7 was highly significant (*p* < 0.001), with stage 7 being larger than stage 6 (*p* < 0.001). Similarly, body width differences between stage 7 and earlier stages (2–6) were also highly significant (*p* < 0.001) (Suppl. material 1: table S5).

As the life cycle progressed, the mean difference between buccal sucker length and width increased. The ANOVA results show that *F* = 60.837 (*p* = 0.000) for the length of the buccal sucker and *F* = 46.522 (*p* = 0.000) for the width of the buccal sucker, indicating significant differences among the groups for the buccal sucker. The results of the LSD test provide additional support for this conclusion. In stage 7, both measurements were significantly greater than in earlier stages (*p* < 0.001). The buccal sucker dimensions in stage 6 were higher than in stages 1–4 (length, *p* < 0.001; width, *p* = 0.001), but still lower than in stage 7 (*p* < 0.001) (Suppl. material 1: table S5).

The ANOVA results show that *F* = 37.761 (*p* = 0.000) for the length of pharynx and *F* = 10.949 (*p* = 0.000) for the width of pharynx, indicating significant differences among the groups for pharynx. The results of the LSD test provide additional support for this conclusion. Pharyngeal length and width increased gradually, with no significant differences in stages 2–5 (*p* > 0.05). Stage 6 showed significant increases in pharyngeal length compared to stages 2 and 3 (between Stage 2 and Stage 6, *p* = 0.009, between Stage 3 and Stage 6, *p* = 0.029), and both dimensions were significantly greater in stage 7 (*p* < 0.001) (Suppl. material 1: table S6).

The ANOVA results show that *F* = 8.676 (*p* = 0.000) for the central hook handle and *F* = 14.090 (*p* = 0.000) for the central hook crochet en fléau, indicating significant differences among the groups for the central hook. The results of the LSD test provide additional support for this conclusion. The length of the central hook handle and crochet en fléau showed significant growth from stage 1 to 2 (*p* < 0.001), but subsequent growth was slower, indicating the most substantial changes occurred early in development (Suppl. material 1: table S6).

The ANOVA results for the length of clamp I analysis showed *F* = 21.550 (*p* = 0.000), and for the width analysis, *F* = 19.209 (*p* = 0.000). For clamp II, the length analysis was *F* = 36.965 (*p* = 0.000), and for the width analysis, *F* = 58.493 (*p* = 0.000). In clamp III, the length analysis was *F* = 69.849 (*p* = 0.000), and the width analysis, *F* = 112.772 (*p* = 0.000). The results of the LSD test provide additional support for this conclusion. Clamp I dimensions showed a gradual increase from stage 1 to 7, with significant differences between most stages. Stage 7 had significantly greater length and width than stages 1–4 (stage 1 to 3, *p* < 0.001; stage 4, *p* = 0.010), but no significant differences from stages 5 and 6 (stage 5, *p* = 0.055; stage 6, *p* = 0.357). For Clamp II, stage 3 had significantly smaller dimensions than stages 4–7 (the length of stage 4, *p* < 0.001, the width of stage 4, *p* = 0.001, stage 5 to 7, *p* < 0.001). No significant differences in Clamp II length were found between stages 4 and 5 (*p* = 0.112), and no significant differences were found between stages 6 and 7 (*p* = 0.267). Clamp III showed significant growth from stage 4 to 6 (length of stage 4 to stage 5, *p* = 0.003, other stages, *p* < 0.010), but growth slowed from stage 6 to 7, with width showing a significant difference (*p* = 0.010), while length did not (*p* = 0.420) (Suppl. material 1: table S7).

In summary, body length and width, oral sucker dimensions, and pharyngeal dimensions all exhibit a gradual increase over time, with significant growth observed between stages 6 and 7. The development of the central hook handle and crochet en fléau shows rapid growth during the transition from the oncomiracidium to the diporpa stage, followed by a slower growth phase. In contrast, the clamps show more continuous growth, reaching a key developmental peak during the adult stage.

## ﻿Discussion

The present study is the first report of *S. coreius* infecting predatory carp and Kanglang fish (both new host records), and first report of *S. coreius* collected from the Lancang River (Nanjian River basin; new locality record). A review of the literature revealed that *S. coreius* is only reported to infect xenocypridids (Cao et al. 2022; 2024); however, these observations are based on a limited number of studies. Therefore, no comment on the host specificity of *S. coreius* should be made until more xenocyprids are examined for diplozoids.

Two eggs were observed herein and compared to those reported in Cao et al. (2022). The eggs observed herein were wider and longer than those of Cao et al. (2022). However, due to the limited sample size, the findings do not meet statistical standards and may be subject to measurement errors. Notably, the filaments of the eggs originate from the operculum, in contrast to those of *E. nipponicum*, where the filaments are located opposite the operculum (Přikrylová et al. 2018). While the eggs mature within the adult *S. coreius*, they exit through the uterine opening located in the fusion area (Cao et al. 2022); however, the egg production process was not directly observed in this study.

Only one oncomiracidium was observed herein. Hodová and Sonnek (2009) reported that the oncomiracidium is the shortest life-history stage in the diplozoid lifecycle (Pečínková et al. 2007) and, upon hatching, must only survive a few hours. Previous studies suggest that cilia serve as flow receptors facilitating movement (Hodová et al. 2010). Although cilia were observed on the oncomiracidium of *S. coreius* in this study, their precise arrangement could not be determined. It is presumed that the arrangement of cilia may be associated with the motility of the oncomiracidium stage (Hodová et al. 2010).

The central hooks reach maximum size in the diporpa. During the oncomiracidium stage, the central hooks play a crucial role in attachment to the gills. Once the larval stage is reached, the clamps take over the attachment function, while the central hooks become non-functional (Khotenovsky 1985).

In this study, all paired worms observed possessed at least three pairs of clamps. This contrasts with the findings of Avenant-Oldewage and Milne (2014), who reported that *Paradiplozoon ichthyoxanthon* Avenant-Oldewage, le Roux, Mashego & van Vuuren, 2013 paired after developing only two pairs of clamps. These developmental differences between *P. ichthyoxanthon* and *S. coreius* could be a generic feature. Two unpaired worms with four pairs of clamps were observed, suggesting that these individuals may not have paired in time. Previous research has indicated that unpaired individuals fail to develop a mature reproductive system (Hodová and Sonnek 2009; Hodová et al. 2010; Valigurová et al. 2011; Jedličková et al. 2019). The morphology of the adult *S. coreius* in this study is consistent with prior descriptions, featuring oval ovaries and testes composed of five small lobes located posterior to the ovaries (Cao et al. 2022).

The body length, size of the four pairs of clamps, and central hooks of seven of our adult specimens were smaller than those of Cao et al. (2022), while the buccal suckers and pharynx of our specimens were larger. This discrepancy may be attributed to the pressure exerted by the cover glass during observation, which likely caused expansion of the buccal suckers and pharynx. In contrast, the haptoral sclerites appeared resistant to such expansion.

## ﻿Conclusions

The study of the life cycle of diplozoids is not only of guiding significance for the prevention and control of parasitic diseases in aquaculture, but also contributes to advances in parasite taxonomy and phylogenetic research. The findings of this study provide the first detailed data on the developmental stages of *S. coreius*, contributing significantly to the understanding of its biology. However, several aspects remain unexplored, including the process of egg production and the surface morphology of the worm. Moreover, current knowledge of the life cycle in the other diplozoid species is limited, and the variations among species and genera have yet to be fully elucidated. These gaps highlight the need for further research to deepen our understanding of diplozoid biology.

## ﻿Additional information

### Use of AI

No use of AI was reported.

### Author ORCIDs

Lu Shen https://orcid.org/0009-0002-5081-4670

Zhuo-Yu Zhao https://orcid.org/0009-0003-7343-3149

Ting Jiang https://orcid.org/0009-0007-8550-7095

Jun-Dong Xu https://orcid.org/0009-0008-4554-0218

Han-Ji Tian https://orcid.org/0009-0004-7697-5426

Ting Jia https://orcid.org/0000-0001-8417-0709

Fei-Yan Meng https://orcid.org/0009-0007-7917-8563

Li-Xian Fan https://orcid.org/0000-0001-9646-0869

### Data availability

The sequence data analyzed in this study consist of ITS2 sequences, including publicly available sequences retrieved from GenBank (accession numbers: DQ098893, DQ098894, DQ098897, KP340974, MT028131, KP340973, OP588755, AJ563372, AF369759, MF460994, AF369761, AF369758, LC517172, DQ098898, MW992745, OL961699, OL961698, OL961697) and newly submitted sequences by the authors (accession numbers: PQ684283, PQ684284). The processed data and the results of statistical analysis are included in the text.

## Supplementary materials

Supplementary material 1 Additional file Data type docx Explanation note **fig. S1.** The agarose gel electrophoresis of ITS2 sequences from two *Sindiplozoon coreius* obtained in this study. **table S1.** List of diplozoid species used for genetic comparison and phylogenetic analysis with ITS2 sequence from *Sindiplozoon coreius*. **table S2.** Collection situation of *Sindiplozoon coreius* in the present research. **table S3.** Summary of morphological and structural measurements of *Sinidiplozoon coreius* at different stages of its life cycle. **table S4.** Pairwise distance (kimura 2-parameter in %) for diplozoids taxa based on the complete ITS2 sequences available in NCBI. **table S5.** The measurement differences of whole body and Oral sucker in different stages of *Sindiplozoon coreius*. **table S6.** The measurement differences of Pharynx and central hooks in different stages of *Sindiplozoon coreius*. **table S7.** Differences of Clamp1,2,3 in different stages of *Sindiplozoon coreius*. https://binary.pensoft.net/file/1456697This dataset is made available under the Open Database License (http://opendatacommons.org/licenses/odbl/1.0/). The Open Database License (ODbL) is a license agreement intended to allow users to freely share, modify, and use this Dataset while maintaining this same freedom for others, provided that the original source and author(s) are credited.Author: Lu Shen
