# Supplementary material for: ﻿Identification of Sindiplozoon coreius (Monogenea, Diplozoidae) and morphological characteristics of the various developmental stages
Source: Zookeys. 2025 Nov 3;1258:137–57. doi: 10.3897/zookeys.1258.162589 (PMC12603642; doi:10.3897/zookeys.1258.162589)
Supplement: Supplementary material 1 — Additional file [file zookeys-1258-137_article-162589__-s001.docx]

**Additional file 1 for:**

**Identification of *Sinidiplozoon coreius* (Monogenea, Diplozoidae) and morphological characteristics of the various developmental stages**

**
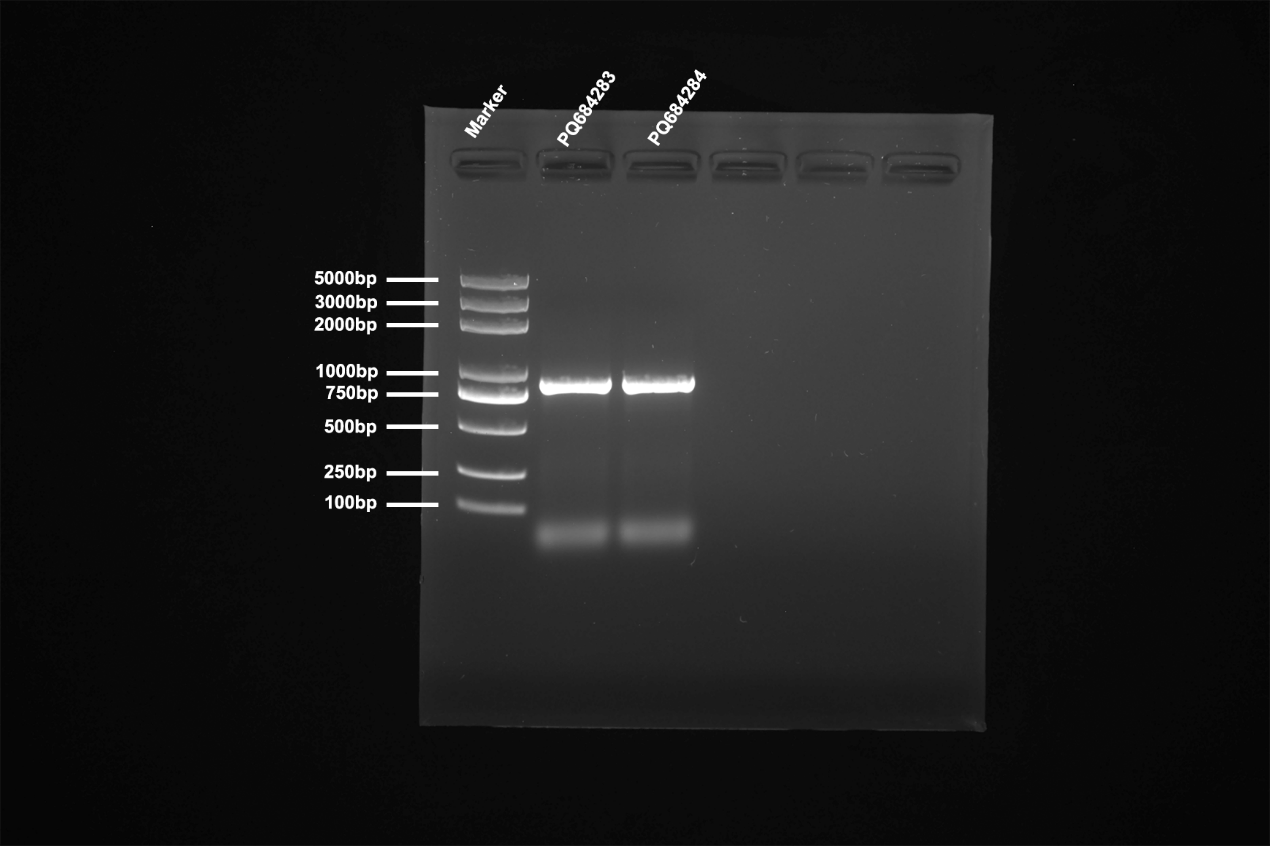
**

**Figure 1.** The agarose gel electrophoresis of ITS2 sequences from two *Sindiplozoon coreius* obtained in this study

**Table 1** List of diplozoid species used for genetic comparison and phylogenetic analysis with *ITS2* sequence from *Sindiplozoon coreius*

| **Parasite species** | **Host species** | **Locality** | **Length (bp)** | **GenBank ID** |
| --- | --- | --- | --- | --- |
| *Inustiatus inustiatus* | *Hypophthalmichthys molitrix* | Tangxun Lake, China | 771 | DQ098893 |
| *I. aristichthysi* | *H. nobilis* | Tangxun Lake, China | 771 | DQ098894 |
| *Eudiplozoon nipponicum* | *Cyprinus carpio* | Danjiangkou Reservoir, China | 804 | DQ098897 |
| *Paradiplozoon skrjabini* | *Leuciscus baicalensis* | China | 764 | KP340974 |
| *P. homoion* | *Rhodeus amarus* | Turkey | 820 | MT028131 |
| *P. gracile* | *Gobio acutipinnatus* | China | 764 | KP340973 |
| *P. bliccae* | *Barbus xanthos* | Turkey | 784 | OP588755 |
| *Diplozoon paradoxum* | *Abramis brama* | Kyjovka River, Czech Republic | 769 | AJ563372 |
| *D. paradoxum*  *D.kashmirensis*  *D. bliccae*  *E.nipponicum*  *E. kamegaii* | *A. brama*  N/A  *Blicca bjoerkna*  *C. carpio*  *C. carpio* | Scamandre pond, Camargue, France  India  France  France  Japan | 1002  848  988  977  625 | AF369759  MF460994  AF369761  AF369758  LC517172 |
| *S. ctenopharyngodoni* | *Ctenopharyngodon idella* | Tangxun Lake, China | 834 | DQ098898 |
| *S. coreius* | *Coreius guichenoti* | Sichuan, China | 722 | MW992745 |
| *S. coreius* | *Schizothorax prenanti* | Hubei, China | 831 | OL961699 |
| *S. coreius* | *Procypris merus* | Guangxi, China | 760 | OL961698 |
| *S. coreius* | *Percocypris pingi* | Hubei, China | 839 | OL961697 |
| *S. coreius* | *Anabarilius grahami* | Yunnan, China | 811 | PQ684283* |
| *S. coreius* | *Cultrichthys erythropterus* | Yunnan, China | 829 | PQ684284* |

*New sequence obtained in the present study.

| Species of host fish | The number of host fish | The total number of parasites | Prevalence ratio | average infection intensity | characteristics | The number of parasites corresponding to the characteristics | The number of clamps | The number of parasites corresponding to the clamps |
| --- | --- | --- | --- | --- | --- | --- | --- | --- |
| *C. erythropterus* | 1 | 8 | 100% | 8 | adult |  |  |  |
| *A. grahami* | 6 | 91 | 100% | 15.17 | X shape | 62 | 4 | 5 |
|  |  |  |  |  |  |  | 4 pairs below | 6 |
|  |  |  |  |  | diporpa | 29 | 1 | 5 |
|  |  |  |  |  |  |  | 2 | 14 |
|  |  |  |  |  |  |  | 3 | 8 |
|  |  |  |  |  |  |  | 4 | 2 |

**Table 2** Collection situation of *Sindiplozoon coreius* in the present research

**Table 3** Summary of morphological and structural measurements of *Sinidiplozoon coreius* at different stages of its life cycle

| stages | | 1 | 2 | 3 | 4 | 5 | 6 | 7 |
| --- | --- | --- | --- | --- | --- | --- | --- | --- |
| Whole body | n | 1 | 5 | 7 | 5 | 4 | 12 | 14 |
|  | Length | 246 | 472±9 | 745±53 | 931±176 | 1075±193 | 2442±313 | 5226±304 |
|  | n | 1 | 4 | 8 | 5 | 4 | 12 | 14 |
|  | width | 90 | 176±15 | 248±24 | 222±24 | 178±24 | 400±52 | 1066±90 |
| Oral sucker | n | 2 | 8 | 12 | 10 | 4 | 10 | 4 |
|  | Length | 27±1 | 38±1 | 46±1 | 49±3 | 51±3 | 70±5 | 139±9 |
|  | n | 2 | 8 | 12 | 10 | 4 | 10 | 4 |
|  | width | 24±1 | 35±1 | 43±2 | 50±3 | 58±3 | 67±5 | 133±13 |
| Pharynx | n | 1 | 2 | 4 | 4 | 2 | 3 | 2 |
|  | Length | 23 | 43±3 | 50±3 | 54±6 | 52±3 | 64±3 | 127±4 |
|  | n | 1 | 2 | 4 | 4 | 2 | 3 | 2 |
|  | width | 27 | 40±1 | 44±4 | 48±5 | 47±4 | 50±1 | 84±5 |
| Central hook | n | 2 | 8 | 12 | 10 | 8 | 22 | 6 |
|  | handle | 28±1 | 49±1 | 46±1 | 44±2 | 48±1 | 47±1 | 50±1 |
|  | n | 2 | 8 | 12 | 10 | 8 | 22 | 6 |
|  | crochet | 12±1 | 22±1 | 20±1 | 19±1 | 22±1 | 20±1 | 24±1 |
| Clamp 1 | n | 2 | 10 | 14 | 10 | 8 | 22 | 12 |
|  | Length | 30±1 | 50±1 | 56±2 | 60±3 | 62±1 | 65±1 | 67±1 |
|  | n | 2 | 10 | 14 | 10 | 8 | 22 | 12 |
|  | width | 39±1 | 69±3 | 77±3 | 81±4 | 88±2 | 91±1 | 94±3 |
| Clamp 2 | n |  |  | 14 | 10 | 8 | 22 | 12 |
|  | Length |  |  | 42±1 | 52±2 | 56±2 | 64±2 | 64±1 |
|  | n |  |  | 14 | 10 | 8 | 22 | 12 |
|  | width |  |  | 59±2 | 73±4 | 87±3 | 101±2 | 105±2 |
| Clamp 3 | n |  |  |  | 10 | 8 | 22 | 12 |
|  | Length |  |  |  | 32±2 | 42±3 | 62±1 | 64±1 |
|  | n |  |  |  | 10 | 8 | 22 | 12 |
|  | width |  |  |  | 45±2 | 62±4 | 99±2 | 108±2 |
| Clamp 4 | n |  |  |  |  |  | 22 | 12 |
|  | Length |  |  |  |  |  | 56±2 | 61±1 |
|  | n |  |  |  |  |  | 22 | 12 |
|  | width |  |  |  |  |  | 85±2 | 94±3 |

All measurements are in μm

n = number of individuals measured for this characteristic (Oral suckers, central hooks and clamps contain measurements of structures on the left and right sides of the body; Stages 5, 6, and 7 include measurement data of the left and right monads of the worm body.)

**Table 4** Pairwise distance (kimura 2-parameter in %) for diplozoids taxa based on the complete *ITS2* sequences available in NCBI.

|  | 1* | 2* | 3 | 4 | 5 | 6 | 7 | 8 | 9 | 10 | 11 | 12 | 13 | 14 | 15 | 16 | 17 | 18 | 19 | 20 |
| --- | --- | --- | --- | --- | --- | --- | --- | --- | --- | --- | --- | --- | --- | --- | --- | --- | --- | --- | --- | --- |
| 1* |  |  |  |  |  |  |  |  |  |  |  |  |  |  |  |  |  |  |  |  |
| 2* | 0.000 |  |  |  |  |  |  |  |  |  |  |  |  |  |  |  |  |  |  |  |
| 3 | 0.000 | 0.000 |  |  |  |  |  |  |  |  |  |  |  |  |  |  |  |  |  |  |
| 4 | 0.000 | 0.000 | 0.000 |  |  |  |  |  |  |  |  |  |  |  |  |  |  |  |  |  |
| 5 | 0.000 | 0.000 | 0.000 | 0.000 |  |  |  |  |  |  |  |  |  |  |  |  |  |  |  |  |
| 6 | 0.000 | 0.000 | 0.000 | 0.000 | 0.000 |  |  |  |  |  |  |  |  |  |  |  |  |  |  |  |
| 7 | 0.044 | 0.044 | 0.044 | 0.044 | 0.044 | 0.044 |  |  |  |  |  |  |  |  |  |  |  |  |  |  |
| 8 | 0.234 | 0.234 | 0.234 | 0.234 | 0.234 | 0.234 | 0.226 |  |  |  |  |  |  |  |  |  |  |  |  |  |
| 9 | 0.234 | 0.234 | 0.234 | 0.234 | 0.234 | 0.234 | 0.226 | 0.000 |  |  |  |  |  |  |  |  |  |  |  |  |
| 10 | 0.351 | 0.351 | 0.351 | 0.351 | 0.351 | 0.351 | 0.343 | 0.129 | 0.129 |  |  |  |  |  |  |  |  |  |  |  |
| 11 | 0.240 | 0.240 | 0.240 | 0.240 | 0.240 | 0.240 | 0.232 | 0.044 | 0.044 | 0.076 |  |  |  |  |  |  |  |  |  |  |
| 12 | 0.240 | 0.240 | 0.240 | 0.240 | 0.240 | 0.240 | 0.229 | 0.044 | 0.044 | 0.081 | 0.004 |  |  |  |  |  |  |  |  |  |
| 13 | 0.212 | 0.212 | 0.212 | 0.212 | 0.212 | 0.212 | 0.209 | 0.069 | 0.069 | 0.164 | 0.072 | 0.072 |  |  |  |  |  |  |  |  |
| 14 | 0.209 | 0.209 | 0.209 | 0.209 | 0.209 | 0.209 | 0.205 | 0.067 | 0.067 | 0.161 | 0.069 | 0.069 | 0.002 |  |  |  |  |  |  |  |
| 15 | 0.209 | 0.209 | 0.209 | 0.209 | 0.209 | 0.209 | 0.205 | 0.067 | 0.067 | 0.161 | 0.069 | 0.069 | 0.002 | 0.000 |  |  |  |  |  |  |
| 16 | 0.334 | 0.334 | 0.334 | 0.334 | 0.334 | 0.334 | 0.327 | 0.292 | 0.292 | 0.369 | 0.281 | 0.274 | 0.282 | 0.278 | 0.278 |  |  |  |  |  |
| 17 | 0.334 | 0.334 | 0.334 | 0.334 | 0.334 | 0.334 | 0.327 | 0.292 | 0.292 | 0.369 | 0.281 | 0.274 | 0.282 | 0.278 | 0.278 | 0.000 |  |  |  |  |
| 18 | 0.342 | 0.342 | 0.342 | 0.342 | 0.342 | 0.342 | 0.343 | 0.299 | 0.299 | 0.377 | 0.288 | 0.282 | 0.290 | 0.286 | 0.286 | 0.008 | 0.008 |  |  |  |
| 19 | 0.488 | 0.488 | 0.488 | 0.488 | 0.488 | 0.488 | 0.484 | 0.443 | 0.443 | 0.567 | 0.440 | 0.445 | 0.446 | 0.441 | 0.441 | 0.388 | 0.388 | 0.392 |  |  |
| 20 | 0.493 | 0.493 | 0.493 | 0.493 | 0.493 | 0.493 | 0.489 | 0.448 | 0.448 | 0.566 | 0.439 | 0.444 | 0.451 | 0.445 | 0.445 | 0.392 | 0.392 | 0.396 | 0.004 |  |

Appellations of 14 taxa are stated below. *New sequence obtained in the present study.

1*. *S. coreius* (PQ684283); 2*. *S coreius* (PQ684284); 3. *S. coreius* (OL961697); 4. *S. coreius* (MW992745); 5. *S.coreius* (OL961698); 6. *S. coreius* (OL961699); 7. *S. ctenopharyngodoni* (DQ098898); 8. *Diplozoon paradoxum* (AF369759); 9. *D. paradoxum* (AJ563372); 10. *D. kashmirensis* (MF460994); 11. *D. bliccae* (AF369761); 12. *P. bliccae* (OP588755); 13. *P. skrjabini* (KP340974); 14. *P. gracile* (KP340973); 15. *P. homoion* (MT028131); 16. *E. nipponicum* (DQ098897); 17. *E. nipponicum* (AF369758); 18. *E. kamegaii* (LC517172); 19. *Inustiatus aristichthysi* (DQ098894); 20. *I. inustiatus* (DQ098893)

**Table 5** The measurement differences of whole body and Oral sucker in different stages of *Sindiplozoon coreius*

|  | | Whole body | | | | | | Oral sucker | | | | | |
| --- | --- | --- | --- | --- | --- | --- | --- | --- | --- | --- | --- | --- | --- |
|  |  | length | | | width | | | length | | | width | | |
| stage（I） | stage(J) | Mean difference（I-J） | Standard error | Statistical significance P | Mean difference（I-J） | Standard error | Statistical significance P | Mean difference（I-J） | Standard error | Statistical significance P | Mean difference（I-J） | Standard error | Statistical significance P |
| 1 | 2 |  |  |  |  |  |  | -11.69 | 7.79 | 0.141 | -10.42 | 8.62 | 0.233 |
|  | 3 |  |  |  |  |  |  | -19.57* | 7.52 | 0.013 | -18.16* | 8.33 | 0.035 |
|  | 4 |  |  |  |  |  |  | -22.57* | 7.63 | 0.005 | -25.37* | 8.45 | 0.004 |
|  | 5 |  |  |  |  |  |  | -24.15* | 8.53 | 0.007 | -33.84* | 9.45 | 0.001 |
|  | 6 |  |  |  |  |  |  | -43.09* | 7.63 | 0.000 | -42.16* | 8.45 | 0.000 |
|  | 7 |  |  |  |  |  |  | -112.50* | 8.53 | 0.000 | -108.97* | 9.45 | 0.000 |
| 2 | 1 |  |  |  |  |  |  | 11.69 | 7.79 | 0.141 | 10.42 | 8.62 | 0.233 |
|  | 3 | -273.52 | 508.46 | 0.594 | -72.08 | 130.98 | 0.585 | -7.88 | 4.49 | 0.087 | -7.74 | 4.98 | 0.127 |
|  | 4 | -459.55 | 549.20 | 0.408 | -46.03 | 143.48 | 0.750 | -10.89* | 4.67 | 0.025 | -14.95* | 5.17 | 0.006 |
|  | 5 | -603.59 | 582.51 | 0.306 | -2.40 | 151.24 | 0.987 | -12.47* | 6.03 | 0.045 | -23.42* | 6.68 | 0.001 |
|  | 6 | -1970.29* | 462.22 | 0.000 | -224.70 | 123.49 | 0.076 | -31.41* | 4.67 | 0.000 | -31.74* | 5.17 | 0.000 |
|  | 7 | -4754.91* | 452.40 | 0.000 | -890.95* | 121.27 | 0.000 | -100.81* | 6.03 | 0.000 | -98.55* | 6.68 | 0.000 |
| 3 | 1 |  |  |  |  |  |  | 19.57* | 7.52 | 0.013 | 18.16* | 8.33 | 0.035 |
|  | 2 | 273.52 | 508.46 | 0.594 | 72.08 | 130.98 | 0.585 | 7.88 | 4.49 | 0.087 | 7.74 | 4.98 | 0.127 |
|  | 4 | -186.03 | 508.46 | 0.716 | 26.06 | 121.94 | 0.832 | -3.01 | 4.22 | 0.480 | -7.21 | 4.67 | 0.130 |
|  | 5 | -330.07 | 544.27 | 0.548 | 69.68 | 130.98 | 0.598 | -4.59 | 5.69 | 0.424 | -15.68* | 6.30 | 0.017 |
|  | 6 | -1696.77* | 412.98 | 0.000 | -152.62 | 97.63 | 0.126 | -23.53* | 4.22 | 0.000 | -24.00* | 4.67 | 0.000 |
|  | 7 | -4481.39* | 401.97 | 0.000 | -818.87* | 94.80 | 0.000 | -92.94* | 5.69 | 0.000 | -90.81* | 6.30 | 0.000 |
| 4 | 1 |  |  |  |  |  |  | 22.57* | 7.63 | 0.005 | 25.37* | 8.45 | 0.004 |
|  | 2 | 459.55 | 549.20 | 0.408 | 46.03 | 143.48 | 0.750 | 10.89* | 4.67 | 0.025 | 14.95* | 5.17 | 0.006 |
|  | 3 | 186.03 | 508.46 | 0.716 | -26.06 | 121.94 | 0.832 | 3.01 | 4.22 | 0.480 | 7.21 | 4.67 | 0.130 |
|  | 5 | -144.04 | 582.51 | 0.806 | 43.62 | 143.48 | 0.763 | -1.58 | 5.83 | 0.788 | -8.48 | 6.45 | 0.196 |
|  | 6 | -1510.74* | 462.22 | 0.002 | -178.67 | 113.85 | 0.124 | -20.52* | 4.40 | 0.000 | -16.79* | 4.88 | 0.001 |
|  | 7 | -4295.35* | 452.40 | 0.000 | -844.93* | 111.43 | 0.000 | -89.93* | 5.83 | 0.000 | -83.61* | 6.45 | 0.000 |
| 5 | 1 |  |  |  |  |  |  | 24.15* | 8.53 | 0.007 | 33.84* | 9.45 | 0.001 |
|  | 2 | 603.59 | 582.51 | 0.306 | 2.40 | 151.24 | 0.987 | 12.47* | 6.03 | 0.045 | 23.42* | 6.68 | 0.001 |
|  | 3 | 330.07 | 544.27 | 0.548 | -69.68 | 130.98 | 0.598 | 4.59 | 5.69 | 0.424 | 15.68* | 6.30 | 0.017 |
|  | 4 | 144.04 | 582.51 | 0.806 | -43.62 | 143.48 | 0.763 | 1.58 | 5.83 | 0.788 | 8.48 | 6.45 | 0.196 |
|  | 6 | -1366.70* | 501.34 | 0.009 | -222.30 | 123.49 | 0.079 | -18.94* | 5.83 | 0.002 | -8.31 | 6.45 | 0.204 |
|  | 7 | -4151.32* | 492.31 | 0.000 | -888.55* | 121.27 | 0.000 | -88.35* | 6.96 | 0.000 | -75.13* | 7.71 | 0.000 |
| 6 | 1 |  |  |  |  |  |  | 43.09* | 7.63 | 0.000 | 42.16* | 8.45 | 0.000 |
|  | 2 | 1970.29* | 462.22 | 0.000 | 224.70 | 123.49 | 0.076 | 31.41* | 4.67 | 0.000 | 31.74* | 5.17 | 0.000 |
|  | 3 | 1696.77* | 412.98 | 0.000 | 152.62 | 97.63 | 0.126 | 23.53* | 4.22 | 0.000 | 24.00* | 4.67 | 0.000 |
|  | 4 | 1510.74* | 462.22 | 0.002 | 178.67 | 113.85 | 0.124 | 20.52* | 4.40 | 0.000 | 16.79* | 4.88 | 0.001 |
|  | 5 | 1366.70* | 501.34 | 0.009 | 222.30 | 123.49 | 0.079 | 18.94* | 5.83 | 0.002 | 8.31 | 6.45 | 0.204 |
|  | 7 | -2784.62* | 341.61 | 0.000 | -666.25* | 84.14 | 0.000 | -69.41* | 5.83 | 0.000 | -66.82* | 6.45 | 0.000 |
| 7 | 1 |  |  |  |  |  |  | 112.50* | 8.53 | 0.000 | 108.97* | 9.45 | 0.000 |
|  | 2 | 4754.91* | 452.40 | 0.000 | 890.95* | 121.27 | 0.000 | 100.81* | 6.03 | 0.000 | 98.55* | 6.68 | 0.000 |
|  | 3 | 4481.39* | 401.97 | 0.000 | 818.87* | 94.80 | 0.000 | 92.94* | 5.69 | 0.000 | 90.81* | 6.30 | 0.000 |
|  | 4 | 4295.35* | 452.40 | 0.000 | 844.93* | 111.43 | 0.000 | 89.93* | 5.83 | 0.000 | 83.61* | 6.45 | 0.000 |
|  | 5 | 4151.32* | 492.31 | 0.000 | 888.55* | 121.27 | 0.000 | 88.35* | 6.96 | 0.000 | 75.13* | 7.71 | 0.000 |
|  | 6 | 2784.62* | 341.61 | 0.000 | 666.25* | 84.14 | 0.000 | 69.41* | 5.83 | 0.000 | 66.82* | 6.45 | 0.000 |

*The significance level for the difference in means is 0.05.

No "post hoc" test was performed for Stage 1 of whole body due to the Oncomiracidium (Stage 1) has only one measurement.

**Table 6** The measurement differences of Pharynx and central hooks in different stages of *Sindiplozoon coreius*

|  | | Pharynx | | | | | | central hooks | | | | | |
| --- | --- | --- | --- | --- | --- | --- | --- | --- | --- | --- | --- | --- | --- |
|  |  | length | | | width | | | handle | | | crochet | | |
| stage（I） | stage(J) | Mean difference（I-J） | Standard error | Statistical significance P | Mean difference（I-J） | Standard error | Statistical significance P | Mean difference（I-J） | Standard error | Statistical significance P | Mean difference（I-J） | Standard error | Statistical significance P |
| 1 | 2 |  |  |  |  |  |  | -20.82* | 3.23 | 0.000 | -10.60* | 1.44 | 0.000 |
|  | 3 |  |  |  |  |  |  | -18.22* | 3.12 | 0.000 | -8.68* | 1.39 | 0.000 |
|  | 4 |  |  |  |  |  |  | -16.26* | 3.16 | 0.000 | -7.00* | 1.41 | 0.000 |
|  | 5 |  |  |  |  |  |  | -20.09* | 3.23 | 0.000 | -9.80* | 1.44 | 0.000 |
|  | 6 |  |  |  |  |  |  | -18.35* | 3.02 | 0.000 | -8.21* | 1.34 | 0.000 |
|  | 7 |  |  |  |  |  |  | -21.95* | 3.33 | 0.000 | -11.75* | 1.49 | 0.000 |
| 2 | 1 |  |  |  |  |  |  | 20.82* | 3.23 | 0.000 | 10.60* | 1.44 | 0.000 |
|  | 3 | -6.83 | 6.23 | 0.296 | -4.13 | 6.08 | 0.511 | 2.60 | 1.86 | 0.168 | 1.92* | 0.83 | 0.024 |
|  | 4 | -10.90 | 6.23 | 0.108 | -7.54 | 6.08 | 0.240 | 4.57* | 1.94 | 0.022 | 3.60* | 0.86 | 0.000 |
|  | 5 | -8.84 | 7.20 | 0.245 | -6.44 | 7.02 | 0.378 | 0.74 | 2.04 | 0.720 | 0.80 | 0.91 | 0.380 |
|  | 6 | -20.64* | 6.57 | 0.009 | -9.17 | 6.40 | 0.180 | 2.47 | 1.69 | 0.148 | 2.40* | 0.75 | 0.002 |
|  | 7 | -84.30* | 7.20 | 0.000 | -43.97* | 7.02 | 0.000 | -1.13 | 2.21 | 0.610 | -1.14 | 0.98 | 0.250 |
| 3 | 1 |  |  |  |  |  |  | 18.22* | 3.12 | 0.000 | 8.68* | 1.39 | 0.000 |
|  | 2 | 6.83 | 6.23 | 0.296 | 4.13 | 6.08 | 0.511 | -2.60 | 1.86 | 0.168 | -1.92* | 0.83 | 0.024 |
|  | 4 | -4.07 | 5.09 | 0.441 | -3.41 | 4.96 | 0.506 | 1.96 | 1.75 | 0.266 | 1.68* | 0.78 | 0.035 |
|  | 5 | -2.00 | 6.23 | 0.754 | -2.31 | 6.08 | 0.711 | -1.87 | 1.86 | 0.321 | -1.12 | 0.83 | 0.183 |
|  | 6 | -13.81* | 5.50 | 0.029 | -5.04 | 5.36 | 0.367 | -0.13 | 1.47 | 0.928 | 0.47 | 0.65 | 0.471 |
|  | 7 | -77.47* | 6.23 | 0.000 | -39.84* | 6.08 | 0.000 | -3.73 | 2.04 | 0.072 | -3.06* | 0.91 | 0.001 |
| 4 | 1 |  |  |  |  |  |  | 16.26* | 3.16 | 0.000 | 7.00* | 1.41 | 0.000 |
|  | 2 | 6.83 | 6.23 | 0.296 | 7.54 | 6.08 | 0.240 | -4.57* | 1.94 | 0.022 | -3.60* | 0.86 | 0.000 |
|  | 3 | -4.07 | 5.09 | 0.441 | 3.41 | 4.96 | 0.506 | -1.96 | 1.75 | 0.266 | -1.68* | 0.78 | 0.035 |
|  | 5 | -2.00 | 6.23 | 0.754 | 1.10 | 6.08 | 0.860 | -3.83 | 1.94 | 0.053 | -2.80* | 0.86 | 0.002 |
|  | 6 | -13.81* | 5.50 | 0.029 | -1.63 | 5.36 | 0.766 | -2.10 | 1.56 | 0.183 | -1.21 | 0.69 | 0.087 |
|  | 7 | -77.47* | 6.23 | 0.000 | -36.43* | 6.08 | 0.000 | -5.70* | 2.11 | 0.009 | -4.75* | 0.94 | 0.000 |
| 5 | 1 |  |  |  |  |  |  | 20.09* | 3.23 | 0.000 | 9.80* | 1.44 | 0.000 |
|  | 2 | 8.84 | 7.20 | 0.245 | 6.44 | 7.02 | 0.378 | -0.74 | 2.04 | 0.720 | -0.80 | 0.91 | 0.380 |
|  | 3 | 2.00 | 6.23 | 0.754 | 2.31 | 6.08 | 0.711 | 1.87 | 1.86 | 0.321 | 1.12 | 0.83 | 0.183 |
|  | 4 | -2.06 | 6.23 | 0.747 | -1.10 | 6.08 | 0.860 | 3.83 | 1.94 | 0.053 | 2.80* | 0.86 | 0.002 |
|  | 6 | -11.80 | 6.57 | 0.100 | -2.73 | 6.40 | 0.678 | 1.73 | 1.69 | 0.308 | 1.59* | 0.75 | 0.038 |
|  | 7 | -75.47* | 7.20 | 0.000 | -37.53* | 7.02 | 0.000 | -1.87 | 2.21 | 0.401 | -1.95 | 0.98 | 0.052 |
| 6 | 1 |  |  |  |  |  |  | 18.35* | 3.02 | 0.000 | 8.21* | 1.34 | 0.000 |
|  | 2 | 20.64* | 6.57 | 0.009 | 9.17 | 6.40 | 0.180 | -2.47 | 1.69 | 0.148 | -2.40* | 0.75 | 0.002 |
|  | 3 | 13.81* | 5.50 | 0.029 | 5.04 | 5.36 | 0.367 | 0.13 | 1.47 | 0.928 | -0.47 | 0.65 | 0.471 |
|  | 4 | 9.74 | 5.50 | 0.104 | 1.63 | 5.36 | 0.766 | 2.10 | 1.56 | 0.183 | 1.21 | 0.69 | 0.087 |
|  | 5 | 11.80 | 6.57 | 0.100 | 2.73 | 6.40 | 0.678 | -1.73 | 1.69 | 0.308 | -1.59* | 0.75 | 0.038 |
|  | 7 | -63.67* | 6.57 | 0.000 | -34.80* | 6.40 | 0.000 | -3.60 | 1.88 | 0.060 | -3.54* | 0.84 | 0.000 |
| 7 | 1 |  |  |  |  |  |  | 21.95* | 3.33 | 0.000 | 11.75* | 1.49 | 0.000 |
|  | 2 | 84.30* | 7.20 | 0.000 | 43.97* | 7.02 | 0.000 | 1.13 | 2.21 | 0.610 | 1.14 | 0.98 | 0.250 |
|  | 3 | 77.47* | 6.23 | 0.000 | 39.84* | 6.08 | 0.000 | 3.73 | 2.04 | 0.072 | 3.06* | 0.91 | 0.001 |
|  | 4 | 73.40* | 6.23 | 0.000 | 36.43* | 6.08 | 0.000 | 5.70* | 2.11 | 0.009 | 4.75* | 0.94 | 0.000 |
|  | 5 | 75.47* | 7.20 | 0.000 | 37.53* | 7.02 | 0.000 | 1.87 | 2.21 | 0.401 | 1.95 | 0.98 | 0.052 |
|  | 6 | 63.67* | 6.57 | 0.000 | 34.80* | 6.40 | 0.000 | 3.60 | 1.88 | 0.060 | 3.54* | 0.84 | 0.000 |

*The significance level for the difference in means is 0.05.

No "post hoc" test was performed for Stage 1 of Pharynx due to the Oncomiracidium (Stage 1) has only one measurement.

**Table 7** Differences of Clamp1,2,3 in different stages of *Sindiplozoon coreius*

|  | | Clamp 1 | | | | | | Clamp 2 | | | | | | Clamp 3 | | | | | |
| --- | --- | --- | --- | --- | --- | --- | --- | --- | --- | --- | --- | --- | --- | --- | --- | --- | --- | --- | --- |
|  |  | length | | | width | | | length | | | width | | | length | | | width | | |
| stage（I） | stage(J) | Mean difference（I-J） | Standard error | Statistical significance P | Mean difference（I-J） | Standard error | Statistical significance P | Mean difference（I-J） | Standard error | Statistical significance P | Mean difference（I-J） | Standard error | Statistical significance P | Mean difference（I-J） | Standard error | Statistical significance P | Mean difference（I-J） | Standard error | Statistical significance P |
| 1 | 2 | -20.17* | 4.31 | 0.000 | -30.18* | 6.89 | 0.000 |  |  |  |  |  |  |  |  |  |  |  |  |
|  | 3 | -26.56* | 4.20 | 0.000 | -38.30* | 6.73 | 0.000 |  |  |  |  |  |  |  |  |  |  |  |  |
|  | 4 | -30.36* | 4.31 | 0.000 | -42.01* | 6.89 | 0.000 |  |  |  |  |  |  |  |  |  |  |  |  |
|  | 5 | -31.71* | 4.40 | 0.000 | -49.07* | 7.04 | 0.000 |  |  |  |  |  |  |  |  |  |  |  |  |
|  | 6 | -34.81* | 4.11 | 0.000 | -51.69* | 6.57 | 0.000 |  |  |  |  |  |  |  |  |  |  |  |  |
|  | 7 | -36.66* | 4.25 | 0.000 | -54.60* | 6.80 | 0.000 |  |  |  |  |  |  |  |  |  |  |  |  |
| 2 | 1 | 20.17* | 4.31 | 0.000 | 30.18* | 6.89 | 0.000 |  |  |  |  |  |  |  |  |  |  |  |  |
|  | 3 | -6.39* | 2.30 | 0.007 | -8.12* | 3.68 | 0.031 |  |  |  |  |  |  |  |  |  |  |  |  |
|  | 4 | -10.20* | 2.49 | 0.000 | -11.83* | 3.98 | 0.004 |  |  |  |  |  |  |  |  |  |  |  |  |
|  | 5 | -11.54* | 2.64 | 0.000 | -18.88* | 4.22 | 0.000 |  |  |  |  |  |  |  |  |  |  |  |  |
|  | 6 | -14.64* | 2.12 | 0.000 | -21.51* | 3.39 | 0.000 |  |  |  |  |  |  |  |  |  |  |  |  |
|  | 7 | -16.49* | 2.38 | 0.000 | -24.41* | 3.81 | 0.000 |  |  |  |  |  |  |  |  |  |  |  |  |
| 3 | 1 | 26.56* | 4.20 | 0.000 | 38.30* | 6.73 | 0.000 |  |  |  |  |  |  |  |  |  |  |  |  |
|  | 2 | 6.39* | 2.30 | 0.007 | 8.12* | 3.68 | 0.031 |  |  |  |  |  |  |  |  |  |  |  |  |
|  | 4 | -3.80 | 2.30 | 0.103 | -3.71 | 3.68 | 0.317 | -10.13* | 2.47 | 0.000 | -14.18* | 3.95 | 0.001 |  |  |  |  |  |  |
|  | 5 | -5.15* | 2.46 | 0.040 | -10.76* | 3.94 | 0.008 | -14.70* | 2.65 | 0.000 | -28.12* | 4.22 | 0.000 |  |  |  |  |  |  |
|  | 6 | -8.25* | 1.90 | 0.000 | -13.39* | 3.04 | 0.000 | -22.42* | 2.04 | 0.000 | -42.22* | 3.26 | 0.000 |  |  |  |  |  |  |
|  | 7 | -10.09* | 2.19 | 0.000 | -16.30* | 3.50 | 0.000 | -22.52* | 2.35 | 0.000 | -46.05* | 3.75 | 0.000 |  |  |  |  |  |  |
| 4 | 1 | 30.36* | 4.31 | 0.000 | 42.01* | 6.89 | 0.000 |  |  |  |  |  |  |  |  |  |  |  |  |
|  | 2 | 10.20* | 2.49 | 0.000 | 11.83* | 3.98 | 0.004 |  |  |  |  |  |  |  |  |  |  |  |  |
|  | 3 | 3.80 | 2.30 | 0.103 | 3.71 | 3.68 | 0.317 | 10.13* | 2.47 | 0.000 | 14.18* | 3.95 | 0.001 |  |  |  |  |  |  |
|  | 5 | -1.35 | 2.64 | 0.611 | -7.05 | 4.22 | 0.099 | -4.56 | 2.83 | 0.112 | -13.93* | 4.52 | 0.003 | -9.66* | 3.03 | 0.003 | -17.28* | 4.49 | 0.000 |
|  | 6 | -4.44* | 2.12 | 0.040 | -9.67* | 3.39 | 0.006 | -12.29* | 2.28 | 0.000 | -28.03* | 3.63 | 0.000 | -29.92* | 2.44 | 0.000 | -53.54* | 3.61 | 0.000 |
|  | 7 | -6.29* | 2.38 | 0.010 | -12.58* | 3.81 | 0.002 | -12.38* | 2.56 | 0.000 | -31.87* | 4.08 | 0.000 | -31.79* | 2.74 | 0.000 | -62.72* | 4.06 | 0.000 |
| 5 | 1 | 31.71* | 4.40 | 0.000 | 49.07* | 7.04 | 0.000 |  |  |  |  |  |  |  |  |  |  |  |  |
|  | 2 | 11.54* | 2.64 | 0.000 | 18.88* | 4.22 | 0.000 |  |  |  |  |  |  |  |  |  |  |  |  |
|  | 3 | 5.15* | 2.46 | 0.040 | 10.76* | 3.94 | 0.008 | 14.70* | 2.65 | 0.000 | 28.12* | 4.22 | 0.000 |  |  |  |  |  |  |
|  | 4 | 1.35 | 2.64 | 0.611 | 7.05 | 4.22 | 0.099 | 4.56 | 2.83 | 0.112 | 13.93* | 4.52 | 0.003 | 9.66* | 3.03 | 0.003 | 17.28* | 4.49 | 0.000 |
|  | 6 | -3.10 | 2.30 | 0.182 | -2.62 | 3.67 | 0.478 | -7.72* | 2.47 | 0.003 | -14.10* | 3.93 | 0.001 | -20.26* | 2.64 | 0.000 | -36.26* | 3.91 | 0.000 |
|  | 7 | -4.94 | 2.54 | 0.055 | -5.53 | 4.06 | 0.177 | -7.82* | 2.73 | 0.006 | -17.94* | 4.35 | 0.000 | -22.13* | 2.92 | 0.000 | -45.44* | 4.32 | 0.000 |
| 6 | 1 | 34.81* | 4.11 | 0.000 | 51.69* | 6.57 | 0.000 |  |  |  |  |  |  |  |  |  |  |  |  |
|  | 2 | 14.64* | 2.12 | 0.000 | 21.51* | 3.39 | 0.000 |  |  |  |  |  |  |  |  |  |  |  |  |
|  | 3 | 8.25* | 1.90 | 0.000 | 13.39* | 3.04 | 0.000 | 22.42* | 2.04 | 0.000 | 42.22* | 3.26 | 0.000 |  |  |  |  |  |  |
|  | 4 | 4.44* | 2.12 | 0.040 | 9.67* | 3.39 | 0.006 | 12.29* | 2.28 | 0.000 | 28.03* | 3.63 | 0.000 | 29.92* | 2.44 | 0.000 | 53.54* | 3.61 | 0.000 |
|  | 5 | 3.10 | 2.30 | 0.182 | 2.62 | 3.67 | 0.478 | 7.72* | 2.47 | 0.003 | 14.10* | 3.93 | 0.001 | 20.26* | 2.64 | 0.000 | 36.26* | 3.91 | 0.000 |
|  | 7 | -1.85 | 2.00 | 0.357 | -2.91 | 3.19 | 0.365 | -0.10 | 2.14 | 0.964 | -3.83 | 3.42 | 0.267 | -1.87 | 2.30 | 0.420 | -9.18* | 3.40 | 0.010 |
| 7 | 1 | 36.66* | 4.25 | 0.000 | 54.60* | 6.80 | 0.000 |  |  |  |  |  |  |  |  |  |  |  |  |
|  | 2 | 16.49* | 2.38 | 0.000 | 24.41* | 3.81 | 0.000 |  |  |  |  |  |  |  |  |  |  |  |  |
|  | 3 | 10.09* | 2.19 | 0.000 | 16.30* | 3.50 | 0.000 | 22.52* | 2.35 | 0.000 | 46.05* | 3.75 | 0.000 |  |  |  |  |  |  |
|  | 4 | 6.29* | 2.38 | 0.010 | 12.58* | 3.81 | 0.002 | 12.38* | 2.56 | 0.000 | 31.87* | 4.08 | 0.000 | 31.79* | 2.74 | 0.000 | 62.72* | 4.06 | 0.000 |
|  | 5 | 4.94 | 2.54 | 0.055 | 5.53 | 4.06 | 0.177 | 7.82* | 2.73 | 0.006 | 17.94* | 4.35 | 0.000 | 22.13* | 2.92 | 0.000 | 45.44* | 4.32 | 0.000 |
|  | 6 | 1.85 | 2.00 | 0.357 | 2.91 | 3.19 | 0.365 | 0.10 | 2.14 | 0.964 | 3.83 | 3.42 | 0.267 | 1.87 | 2.30 | 0.420 | 9.18* | 3.40 | 0.010 |

*The significance level for the difference in means is 0.05.

No "post hoc" test was performed for clamp 4 due to the number of groups being less than three.
